# Acute Psychosis Presenting With Dengue Fever Complicated by Dengue Encephalitis

**DOI:** 10.7759/cureus.55628

**Published:** 2024-03-06

**Authors:** Hassan Hussain, K.V.C. Janaka, Harsha Gunasekara, Manojkumar Krishnan, Isuru Perera

**Affiliations:** 1 General Medicine, Sri Jayewardenepura General Hospital, Colombo, LKA; 2 Internal Medicine, Sri Jayewardenepura General Hospital, Colombo, LKA; 3 Neurology, Sri Jayewardenepura General Hospital, Colombo, LKA; 4 Medicine, Sri Jayewardenepura General Hospital, Colombo, LKA

**Keywords:** dengue hemorrhagic fever, thrombocytopenia, flavivirus, altered level of consciousness, dengue encephalitis

## Abstract

Dengue is an infection with a wider spectrum of disease manifestations, ranging from simple dengue fever to expanded dengue syndrome. Expanded dengue syndrome encompasses multiorgan involvement, including neurological manifestations such as dengue encephalitis, seizures, encephalopathy, coma, hemiparesis, etc. Herein, we present a case of a 50-year-old female with a background history of well-controlled type 2 diabetes mellitus and hypertension for five years on oral medication. The patient presented with a one-day history of altered levels of consciousness, agitation, and aggressive behavior. Before admission, she had a history of high-grade fever with chills and rigors for three days. Serial investigations were performed, and the diagnosis of dengue encephalitis was made amidst the absence of positive findings for encephalitis in most of the imaging modalities except in electroencephalogram (EEG), making this case unique. Initially, it was presumed to be meningoencephalitis. Hence, the patient was initiated on intravenous acyclovir and cefotaxime. After the definitive diagnosis of dengue encephalitis, the given medication was stopped after seven days of administration, and with supportive management, the patient made a successful recovery within 10 days.

## Introduction

Dengue is a viral infection caused by a flavivirus [1]. Four major serotypes of flavivirus are identified as causative organisms for dengue infection: DEN-1, DEN-2, DEN-3, and DEN-4 [1]. These viruses are transmitted by the mosquitos *Aedes aegypti* and *Aedes albopictus*. The greatest disease burden is noted in the Southeast Asian region [1]. The serotypes DEN-2 and DEN-3 are found to be classically involved in causing neurological complications of dengue [2]. In dengue, the involvement of the central nervous system is due to the neurotropic nature of the virus, and it leads to many neurological disease manifestations, encompassing encephalitis, meningitis, stroke, and, in inevitable cases, death [1].

## Case presentation

A 50-year-old female patient with a background history of well-controlled type 2 diabetes mellitus and hypertension on oral medications presented with a one-day history of agitation, altered level of consciousness, and aggressive behavior. It was of gradual onset, which progressively worsened over time. Three days before the onset of these symptoms, she experienced a high-grade fever accompanied by chills and rigors, generalized body aches, arthralgia, and myalgia. Additionally, she had a continuous diffuse headache of moderate severity, for which she did not seek any medical advice, as the fever responded to simple analgesics. She denied having nausea, vomiting, or abdominal pain, and apart from the aforementioned symptoms, her systemic inquiry was unremarkable. There was no history of seizures, and she denied any history of trauma to the head, ingestion of poisons, or drug overdose. She did not have a history of similar presentations before this admission. There was no history of any weakness in limbs or any visual disturbances. She did not have postural dizziness, reduced urine output, or bleeding tendencies at the time of admission. Apart from the aforementioned medical conditions, her past medical and surgical histories were unremarkable. She had no known allergies. She did not have any significant recent travel history or muddy water exposure.

Upon examination, she was an average-built female whose appearance was consistent with her chronological age. At the time of admission, she was restless and agitated and was not oriented to time, place, and person. Her Glasgow Coma Scale (GCS) was 13/15 (E-4, V-4, and M-5). She was afebrile, and no conjunctival pallor or plethora was noted. No rashes, limb weakness, or ophthalmoplegia was present. Fundal examination was normal, and no signs of meningism were present.

Her pulse rate was 84 beats per minute, regular, and normal in character. Peripheral pulses were present without any delays. The rest of the vitals were stable with a blood pressure of 130/90 mmHg and a capillary refilling time of <2 s. First and second heart sounds were heard with no murmurs. Neurological examination was unremarkable with no gaze palsies, and pupils were equal and reactive to light. Her cranial nerves were intact, and no abnormality was noted in motor and sensory components. There was no right hypochondrial tenderness, and the rest of the systemic examination was unremarkable.

Upon admission, a random blood glucose analysis was conducted, revealing a level of 160 mg/dL, with an HbA1C of 7%. Multiple investigations were conducted to reach a diagnosis, including infective screening and other basic investigations (Tables 1-2).

**Table 1:** Infective screening. NS1, non-structural protein 1; IgM, immunoglobulin M; CSF, cerebrospinal fluid; RT-PCR, reverse transcription polymerase chain reaction; EBV, Epstein-Barr virus; HPF, high-power field

Investigation		Results
Infective screening	Dengue NS1 antigen	Positive
	Dengue IgM antibody (in blood and CSF) and dengue IgG antibody - in blood	Positive
	Dengue PCR - from CSF	Negative
	COVID-19 RT-PCR	Negative
	Varicella-specific IgM antibody	Negative
	Cytomegalovirus IgM antibody	Negative
	EBV IgM antibody	Negative
	Herpes simplex virus 1 and 2 polymerase chain reaction (HSV PCR)	Negative
	Leptospirosis PCR	Negative
	Urine full report	Pus cells: 2-3/HPF red cells - nil
	Blood and urine culture	No growth yielded
	Cerebrospinal fluid culture	No growth yielded
	Cerebrospinal fluid analysis	Appearance - colorless/clear glucose - 202 mg/dL (random blood sugar - 377 mg/dL), protein - 41.7 white blood cell count - nil

**Table 2:** Investigations.

Investigations		Values					Reference range
		D1	D2	D3	D5	D7	
Full blood count	White cell count (*10^3^)	5.7	2.61	2.25	4.74	7.74	4-11
	Neutrophils (%)	75	72.4	47.3	32	52	50-70
	Hemoglobin (g/dL)	13.3	12.7	13.3	13.7	12.1	12-14
	Hematocrit (%)	38	36	39.7	41	37	37-54
	Platelets (*10^3^/µL)	182	138	110	38	227	150-400
Liver biochemistry	Alanine transaminases (U/L)	120	-	-	241	149	<40
	Aspartate transaminases (U/L)	99.4	-	-	300	101	<40
	Alkaline phosphatase (U/L)	102.8	-	-	162	173	30-120
	Total bilirubin (mg/dL)	0.2	-	-	0.6	0.6	0.3-1.2
	Gamma-glutamyl transferase (U/L)	87	-	-	-	-	5-40
	Serum albumin (g/dL)	3.5	-	-	-	-	3.4-5.4
	Serum albumin-to-globulin ratio	1.3	-	-	-	-	1-2
Inflammatory markers	C-reactive protein (mg/L)	23	-	-	17	-	<5
	Erythrocyte sedimentation rate (mm in the first hour)	16	-	-	-	-	<20
Serum electrolytes	Serum sodium (mmol/L)	140	-	-	135	-	135-145
	Serum potassium (mmol/L)	3.1	-	-	3.6	-	3.5- 5.5
	Serum magnesium (mmol/L)	0.7	-	-	-	-	
	Serum calcium (mg/dL)	8.4	-	-	-	-	8.6-10
Renal function tests	Serum creatinine (µmol/L)	67	-	-	50	-	45-90
	Blood urea (mg/dL)	27	-	-	-	-	15-40

Meanwhile, imaging studies were conducted to confirm the diagnosis of dengue encephalitis, but only EEG findings were compatible with the postulated diagnosis (Table 3). A lumbar puncture was also conducted, and cerebrospinal fluid (CSF) analysis was performed. The results indicated normal protein and glucose levels, with no pleocytosis, and the absence of oligoclonal bands. Gram staining and culture of the CSF did not reveal the presence of any organisms.

**Table 3:** Imaging studies.

Investigation		Results
Imaging	Magnetic resonance imaging (MRI) Brain	Essentially negative contrast study except for two foci of subcortical hyperintensities in the left frontoparietal region, which can represent ischemic changes
	Non-contrast-enhanced computed tomography (NCCT) brain	Normal study
	Electroencephalogram (EEG)	Intermittent bursts of theta activity and left focal sharp wave activity were noted, consistent with meningoencephalitis.

She was initially stabilized with 5 mg of intramuscular haloperidol in the acute setting. Given her origin from an area with a higher number of reported dengue cases and a history of fever for the past three days before admission, screening for dengue infection was conducted, revealing a positive result for dengue NS1 antigen. This occurred against the backdrop of initially lower-than-normal platelet counts. She was managed for dengue fever with hydration and symptomatic treatment. From day 8 of the onset of the fever, her platelets started to drop and her hematocrit (HCT) started rising, with a bedside ultrasound scan showing signs of fluid leakage, after which a diagnosis of dengue hemorrhagic fever was made.

She was managed with strict fluid maintenance and was started on empirical cefotaxime 1 g and intravenous acyclovir 10 mg/kg/body weight every eight hours from day 1 of admission. The presumptive diagnosis of meningoencephalitis was later ruled out as blood and urine cultures did not reveal any growth. Later a definitive diagnosis of dengue encephalitis was made; hence, the medication was stopped after seven days of administration. Within 10 days of in-hospital stay, her clinical status improved dramatically, after which she was discharged.

## Discussion

Among the viral infections that invade the human immune system, the most common arthropod-borne viral infection is dengue. The flavivirus that is responsible for this infection has a single-stranded, enveloped ribonucleic acid [3]. The spectrum of disease manifestation in dengue can range from asymptomatic dengue infection to symptomatic illness, which can be further classified as dengue fever, dengue hemorrhagic fever, and complicated dengue infections involving various organ systems [4].

Considering the systemic involvement in dengue, 4% to 21% of dengue cases present with encephalitis or meningoencephalitis, as seen in this particular case [5]. Encephalitis occurs as a result of inflammation in the brain parenchyma due to viral invasion, often stemming from the disruption of the blood-brain barrier. Differentiating dengue infections from other central nervous system infections that also cause encephalitis with a similar presentation is challenging because of the shared neurotropic nature of different viruses [5]. Accordingly, viral studies for cytomegalovirus (CMV), Epstein-Barr virus (EBV), varicella-zoster virus, and herpes simplex virus (HSV) were performed in this case to exclude these infections, as they are neurotropic viruses that can lead to a similar clinical presentation [6]. But in this particular case, all were found to be negative. Japanese encephalitis, enterovirus, and coxsackievirus could have presented a similar clinical picture due to their neurotropic nature. However, due to financial constraints and the unavailability of these investigations in the government sector, they could not be conducted for this patient.

The usual clinical manifestations of dengue encephalitis occur in the viremic phase of the illness due to direct invasion of the virus. This was classically seen in this case as well. In the majority of reported cases of dengue encephalitis, the cardinal symptoms that were present include headache, seizures, hemiparesis, and coma, but none of them were evident in our case except for acute onset behavioral changes [7,8].

Literature suggests three clinical criteria that could be advocated to define dengue encephalitis in the clinical setting [9]. They include detecting clinical signs and symptoms of neurological involvement; identifying the presence of viral RNA, IgM, or positive NS1 antigen in CSF; and observing CSF pleocytosis in the absence of other neuroinvasive organisms [9]. But still, there is evidence that 75% of cases of dengue encephalitis can present with normal cellularity noted in CSF analysis [10]. As our patient was acutely disturbed at the time of presentation, CSF studies could not be done; hence, the lumbar puncture was performed during the recovery phase when the patient was more cooperative. During the recovery phase, CSF pleocytosis was not present in this patient and no neuroinvasive pathogen was detected either. However, the delay in performing CSF studies did not hinder the management process as supportive evidence could be obtained from peripheral blood, which showed positive dengue NS1 antigen even on day 4 of fever. The presence of dengue antibodies in CSF can be used as strong evidence to suggest a direct invasion of the dengue virus into the central nervous system [1]. But again, this was not possible in this case due to the inability to perform lumbar puncture at the initial presentation.

The risk factors that are known to contribute to the neurological involvement in dengue are thrombocytopenia, raised HCT, hepatic dysfunction, and high-grade fever [11]. In our patient, high-grade fever was present from day 1 of infection, but the other features, including thrombocytopenia and hepatic dysfunction, were not present in this patient until day 8 of infection, which makes this case atypical from the usual clinical pattern. In the initial evaluation, she had no signs of bleeding manifestation, and there was also an absence of right hypochondrial tenderness. These factors made the clinician think of the possibility of dengue encephalitis as a differential diagnosis for the aforementioned presentation. During the illness, the elevation in liver enzymes was not markedly high and ranged slightly above 3 times the upper limit of normal. This finding did not align with the known risk factors observed as causative factors for the development of dengue encephalitis.

Considering the imaging modalities, the majority of cases of dengue encephalitis show changes in MRI and/or CT. They are seen as hyperintensities/increased density involving bilateral parietal regions, corona radiata, basal ganglia, thalamus, or internal capsule [12]. But in this case, both MRI and CT were found to be essentially negative, making the diagnosis of dengue encephalitis inconclusive at the initial stages. The major disadvantage of the imaging studies in confirming the diagnosis is that none of the investigations show a uniform pattern; hence, diagnosis of this condition based on imaging modalities per se can be ineffective [1]. However, they are useful in excluding other intracranial pathologies such as hemorrhage/infarction, which could have presented with a similar clinical presentation. Hence, it's imperative to continue studies to evaluate the sensitivity and specificity of each of these investigations to improve treatment outcomes.

## Conclusions

The incidence of dengue infection is exponentially rising in various parts of the world, especially in the Southeast Asian region. Unlike in the usual setting, this patient did not show typical findings in the usual imaging studies that brought about a diagnostic dilemma at the initial stages. Even thrombocytopenia, which is classically noted in dengue, was not apparent in this case until the latter half of the disease. Hence, a broader knowledge about different atypical findings is of paramount importance in making an early diagnosis and prompt intervention. 
